# Conservative management (marsupialization) of unicystic ameloblastoma: literature review and a case report

**DOI:** 10.1186/s40902-017-0134-0

**Published:** 2017-12-25

**Authors:** Jwayoung Kim, Eunkyung Nam, Sukho Yoon

**Affiliations:** Department of Oral and Maxillofacial Surgery, Hallym University Medical Center, Hallym University Kangnam Sacred Heart Hospital, Seoul, Republic of Korea

**Keywords:** Unicystic ameloblastoma, Conservative treatment, Marsupialization, Tooth eruption

## Abstract

**Background:**

In this study, we present a case of unicystic ameloblastoma (UA) treated by marsupialization followed by surgical enucleation as a conservative approach. UA is a rare, benign, less aggressive, and less invasive variant of ameloblastoma that is observed quite often in younger patients. Radical approaches have effects on the physical and psychological development of a growing young patient; therefore, conservative approaches are widely used for UA management in children.

**Case presentation:**

This report described a case of an 11-year-old girl with UA of the mandibular molar–ramus area, which also involved impaction of the second and third molars. The lesion was marsupialized, and 31 months after marsupialization, surgical enucleation was performed with extraction of the impacted third molar. The second molar, which was preserved, spontaneously and completely erupted. No recurrence was observed during a 49-month follow-up.

**Conclusions:**

Conservative treatments for UA in young patients have more advantages. Our results provide evidence that marsupialization is effective in regressing the lesion size to ease complete removal, preserving mandibular growth, maintaining involved second molar, and promoting the eruption of the tooth.

## Background

Ameloblastoma is a rare, benign odontogenic tumor that manifests locally as an aggressive neoplasm of the jaw, developing from the epithelium involved in the formation of teeth: the enamel organ, epithelial cell rests of Malassez, reduced enamel epithelium, and odontogenic cyst lining [1–3]. It occurs in the maxilla and mandible but is most prevalent in the mandible [4, 5]. Ameloblastoma is slow growing, usually asymptomatic, and is found during routine dental radiographs. However, this condition may also present with jaw expansion [4, 6]. On the basis of the histologic architecture, clinical behavior, and prognosis, four types of ameloblastomas can be classified: (1) conventional or classical, intraosseous, solid, or multicystic ameloblastoma; (2) unicystic ameloblastoma (UA); (3) peripheral or extraosseous ameloblastoma; and (4) desmoplastic ameloblastoma [1, 2, 7, 8]. Of these, 5 to 15% of all ameloblastomas are of the unicystic type [7].

UA presents some distinguishing and characteristic features because it is observed in a rather younger age group [1, 8, 9] than conventional ameloblastoma, which is rarely observed in younger populations [10, 11]. Radiographically, the unilocular pattern is more common than the multilocular pattern, particularly in cases associated with tooth impaction [1, 8]. Impacted mandibular third molars are even frequently associated with UA [8]. It also usually appears very similarly to a non-neoplastic odontogenic cyst and is frequently misdiagnosed as a dentigerous cyst or an odontogenic keratocyst. Therefore, histologic confirmation with biopsy is mandatory [9, 11, 12]. UA is believed to be less aggressive, and it responds more favorably to conservative treatments than the multicystic or solid types [1, 5, 6].

Various treatment modalities such as segmental or marginal resectioning for UA have been used, similar to those normally used for conventional ameloblastoma, and more conservative treatments have also been frequently reported [9, 13]. The traditional treatment of a complete resection of the lesion site could result in numerous complications, such as functional and masticatory change, mutilations, and facial deformities [14]. To avoid complications associated with more radical operations, conservative treatments are widely employed for treating UA in children [1, 2, 9, 12, 15, 16].

This study presents a case of mandibular UA in a young patient who was successfully treated by conservative management without the removal of an involved second molar.

## Case presentation

In January 2013, an 11-year-old female patient with a chief report of swelling in the left mandibular molar region was referred to our medical center. Two weeks before the first visit, the patient was prescribed antibiotics by a local family medical clinic because of swelling in the left side of the jaw and tenderness of the mandibular angle. For these reasons, she was referred from a local dental clinic and consulted our center. The patient had no systemic health conditions. Extraoral examination revealed swelling in the left mandibular angle area, and intraoral examination revealed mucosal swelling that extended from the lower first molar to the retromolar region, mixed dentition, and an unerupted left mandibular second molar.

**Fig. 1:**
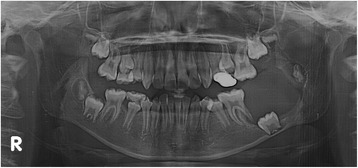
A panoramic radiograph before marsupialization at the initial visit revealing a well-circumscribed, unilocular radiolucency in the region of the left mandibular molars, with an unerupted second molar and dental follicle of the third molar

**Fig. 2:**
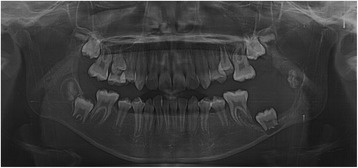
One month after marsupialization

**Fig. 3:**
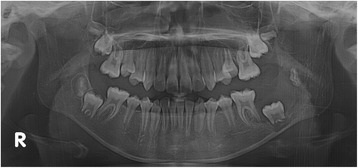
Three months after marsupialization

**Fig. 4:**
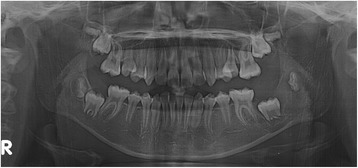
Six months after marsupialization

**Fig. 5:**
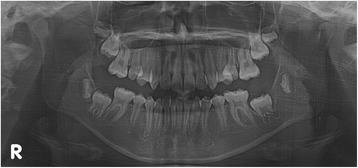
Twelve months after marsupialization

**Fig. 6:**
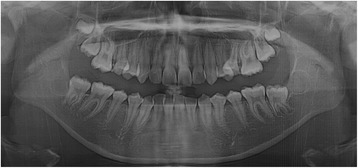
Thirty-one months after marsupialization

**Fig. 7:**
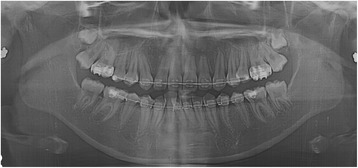
Forty-eight months after marsupialization

A panoramic radiograph revealed a well-circumscribed, unilocular radiolucency in the region of the left mandibular molars, extending from the distal root of the first molar area to the left ascending ramus, with an unerupted second molar, and the dental follicle of the third molar (Fig. 1). The impacted left mandibular second molar presented with incomplete root formation and displacement up to the inferior border of the body. The left mandibular third molar was located on the coronoid process of the mandible. Considering the clinical and radiographic findings, a provisional differential diagnosis of UA, dentigerous cyst, and odontogenic keratocyst was considered. With the patient under local anesthesia, an incisional biopsy was conducted; after histopathologic evaluation, the lesion was diagnosed as UA. The parents were informed of the condition, proposed treatment, recurrence, and benefits, after which they provided their informed consent for the conservative treatment. Marsupialization of the gingiva and cystic wall was performed using a silastic drain to maintain continuity between the marsupialized lesion and oral environment and to ultimately reduce the lesion size. The patient was scheduled for follow-up and daily management for the first month, followed by weekly management. The parents were instructed to maintain overall proper hygiene of the oral cavity through self-irrigation after marsupialization. After 1 month, a propensity of the lower left second molar toward eruption was observed (Fig. 2). After confirmation of epithelization of the cystic wall, the drain was removed. Three months after marsupialization, the lesion diminished in size, new bone formation was observed, and the lower left second molar was in a more advanced phase of eruption (Fig. 3). Radiographs obtained 6 months after marsupialization showed that the lesion margin had lost clarity and that the regenerated bone was replaced by normal trabeculae. The radiolucent area was significantly reduced (Fig. 4). Twelve months after marsupialization, a part of the lower left second molar was observed in the oral cavity (Fig. 5). Thirty months after marsupialization, the lower left second molar erupted on the same occlusal plane as the lower right second molar. The lower left third molar was impacted. In the following month, enucleation of the lesion was performed to completely remove the lesion along with the impacted third molar with the patient under general anesthesia (Fig. 6). The second molar was preserved. The patient began orthodontic treatment for reduction of mild crowding. At 36 months after marsupialization, radiographs showed complete eruption of the lower left second molar, whereas a mild interdental space was observed between the first and second molars. At 48 months after marsupialization, complete occlusion was observed. This tooth was eventually preserved and did not require a root canal (Fig. 7). To date, no evidence of tumor recurrence has been observed on examinations during the 49-month follow-up.

## Conclusions

The treatment of UA has been controversial and can be radical or conservative [1, 2, 9, 15, 17–19]. Radical approaches can be achieved by segmental or marginal resectioning of the lesion, followed by insertion of reconstructive plates [1, 9]. Conversely, conservative treatments comprise enucleation, enucleation followed by application of Carnoy’s solution, or marsupialization followed by enucleation [1, 2, 9, 16, 20]. No adequate evidence proves which treatment modality is the most effective, and many reasons exist for this practical variability and controversy. Because of the relative rarity of this tumor, a definitive conclusion for this debate is difficult to reach [9].

One of the factors that can determine the effectiveness of a treatment is the recurrence rate, which varies with the different types of ameloblastoma [17]. UA is less aggressive than the solid type but has the potential for recurrence [19]. The reported recurrence rate after treating UA ranges from 10 to 25% [9]. According to many studies, the recurrence rate after radical treatment is lower than that after conservative treatment [1, 4, 9, 19, 21]. According to Lau and Samman, Enucleation alone yielded the highest recurrence rate among treatments (30.5%), while the lowest (3.6%) was observed with resection [9]. Seintou et al. reported a recurrence rate of 29.4% after enucleation or excision, and all recurrent cases were related to the conservative approach with enucleation or excision [1]. No recurrence was observed after resection. Sampson and Pogrel reviewed the records of 26 patients referred for management of mandibular ameloblastoma and reported that all patients treated with curettage alone developed recurrence [4].

It is clear that if an adequate bone margin is removed, the chances of recurrence are expected to be low. However, a balanced judgment is required when selecting this treatment option so that maximum success does not lead to overtreatment [9]. Despite the low recurrence rates of resection, there are reasons why radical resectioning of an ameloblastoma in children is often avoided [17]. The treatment of UA in children is complicated because of three factors: (1) continuing facial growth and different bone physiology (more cancellous bone, increased bone turnover, and reactive periosteum), (2) presence of unerupted teeth, and (3) difficulty in the initial diagnosis [1]. Radical surgery is associated with deformity, dysfunction, and numerous complications, including removal of teeth, masticatory dysfunction, and abnormal jaw movement, even after successful reconstruction. Particularly in young patients in the developmental period, the lack of mandibular growth can cause severe facial deformity, which directly influences quality of life [1, 2, 9, 15, 16]. Tanaka et al. demonstrated that minimal surgical treatment should be the first choice procedure for any case of oral and maxillofacial benign tumors in children [14]. Therefore, despite a high success rate for the resection of UA, more conservative treatments have been recently favored [6, 12, 13, 20, 22, 23].

UA’s biologic behavior is considered to be less invasive, and it responds more favorably to conservative treatment than a multicystic ameloblastoma [18]. Therefore, conservative therapy was performed in our patient. We used marsupialization as an initial treatment of UA to minimize the tumors’ volume, and after they regressed in size, they were enucleated.

The aim of marsupialization is to reduce the size of the tumor so that a less extensive surgery is required [23, 24]. The decompression of the internal contents by marsupialization promotes remodeling of bone and osteogenesis [2, 9]. The benefits of decompression are maintenance of pulp vitality, preservation of the inferior alveolar nerve or maxillary sinus, preservation of the mandibular contour and growth, prevention of fracture of the jaw, and low risk for recurrence [20, 25]. According to a systematic review by Lau and Samman, when marsupialization was performed with or without further treatment, the recurrence rate (18%) was lower than that of enucleation alone (30.5%) [9]. The outcome of marsupialization is affected by various factors such as age, technique of marsupialization, removal of solid growths during incisional biopsy, close radiographic follow-up, and effectiveness of enucleation after marsupialization [23]. Nakamura et al. who evaluated the effectiveness of marsupialization for cystic ameloblastomas, concluded that marsupialization was useful as a preliminary treatment of the tumor [16, 20]. They further reported that the effect of marsupialization mainly depends on the following factors: (1) potential for new bone formation, (2) technique of marsupialization, and (3) growth characteristics of the tumor. The potential for bone formation is mainly influenced by the patient’s age. Marsupialization appears to be more effective in young patients, particularly those in the second decade of life. In contrast, lesions could not be easily decompressed in patients who were aged > 60 years. Accordingly, a longer time is required for marsupialization to be effective in older patients [16].

During surgery, the involved teeth are usually extracted together with the tumors to not only completely remove the tumors, but also prevent their recurrence. However, tooth loss can result in functional and esthetic disturbances. Furthermore, prosthodontic treatments are generally difficult in young adolescents because of their dental and skeletal growth. If impacted or involved teeth within ameloblastomas can be preserved and if functional occlusion can be obtained, the patients’ quality of life will significantly improve, particularly in young patients [7].

Marsupialization therapy is usually performed for preserving the involved teeth and promoting eruption of the tooth within the lesion [7, 26, 27]. Hyomoto et al. found that the eruption potential is closely related to root formation [26]. An impacted tooth with an incomplete root and with an open apex has a considerable potential to erupt. Sano et al. reported the spontaneous eruption of an involved second molar in UA of the mandible after marsupialization followed by enucleation and believed that spontaneous eruption and favorable occlusion were obtained for four reasons [7]. First, the lesion was histologically diagnosed as a tumor without infiltration into the surrounding bone. Second, marsupialization reduced the cavity of the unicystic tumor. Third, the impacted second molar was in a condition without complete root formation and with open apices. Fourth, the second molar, rather than being adjacent to the other teeth, had sufficient eruption space.

In our case, the eruption of the mandibular second molar on the affected side was disturbed by the tumor. The impacted second molar naturally erupted after reducing the tumor volume from marsupialization. We eventually decided to keep the second molar, although the third molar had to be extracted.

Conservative treatment appears to be preferable in the younger age groups because it offers a better quality of life; however, the recurrence rate remains high [1]. Therefore, during surgical treatment after marsupialization, careful attention should be given to removing the tumor by sufficient curettage of surrounding tissues [16]. Moreover, long-term follow-up is important for conservative treatment of UA because > 50% of recurrences occur within 5 years of the treatment [1, 19]. Scariot et al. also believe that a more aggressive surgical approach should be considered when the condition recurs more than twice or when required by the patient [17]. Tanaka et al. argue that in cases of recurrence, a second surgery should be more extensive, but overtreatment should be avoided in children [14].

In conclusion, conservative treatment for UA in young patients has more advantages. Our young patient who was treated by conservative management did not have any complications, and the condition was well maintained, with no signs of recurrence. Spontaneous eruption occurred after marsupialization without the extraction of the involved second molar. This tooth functions well without the need for a root canal.

## Abbreviations

UA: Unicystic ameloblastoma

## References

[1] Seintou A, Martinelli-Kläy CP, Lombardi T (2014). Unicystic ameloblastoma in children: systematic review of clinicopathological features and treatment outcomes. Int J Oral Maxillofac Surg.

[2] De Melo WM, Pereira-Santos D, Sonoda CK, Pereira-Freitas SA, de Moura WL, de Paulo Cravinhos JC (2012). Large unicystic ameloblastoma of the mandible: management guided by biological behavior. J Craniofac Surg.

[3] Rapidis AD, Andressakis DD, Stavrianos SD, Faratzis G, Arnogiannaki-Liappi N, Lagogiannis GA, Apostolikas N (2004). Ameloblastomas of the jaws: clinico-pathological review of 11 patients. Eur J Surg Oncol.

[4] Sampson DE, Pogrel MA (1999). Management of mandibular ameloblastoma: the clinical basis for a treatment algorithm. J Oral Maxillofac Surg.

[5] Bisinelli JC, Ioshii S, Retamoso LB, Moysés ST, Moysés SJ, Tanaka OM (2010). Conservative treatment of unicystic ameloblastoma. Am J Orthod Dentofac Orthop.

[6] Paikkatt VJ, Sreedharan S, Kannan VP (2007). Unicystic ameloblastoma of the maxilla: a case report. J Indian Soc Pedod Prev Dent.

[7] Sano K, Yoshimura H, Tobita T, Kimura S, Imamura Y (2013). Spontaneous eruption of involved second molar in unicystic ameloblastoma of the mandible after marsupialization followed by enucleation: a case report. J Oral Maxillofac Surg.

[8] Philipsen HP, Reichart PA (1998). Unicystic ameloblastoma. A review of 193 cases from the literature. Oral Oncol.

[9] Lau SL, Samman N (2006). Recurrence related to treatment modalities of unicystic ameloblastoma: a systematic review. Int J Oral Maxillofac Surg.

[10] Al-Khateeb T, Ababneh KT (2003). Ameloblastoma in young Jordanians: a review of the clinicopathologic features and treatment of 10 cases. J Oral Maxillofac Surg.

[11] Kim SG, Jang HS (2001). Ameloblastoma: a clinical, radiographic, and histopathologic analysis of 71 cases. Oral Surg Oral Med Oral Pathol Oral Radiol Endod.

[12] Kalaskar R, Unawane AS, Kalaskar AR, Pandilwar P (2011). Conservative management of unicystic ameloblastoma in a young child: report of two cases. Contemp clin dent.

[13] de Paulo LFB, Oliveira MTF, Rodrigues ÁR, Zanetta-Barbosa D (2015). Treatment of an extensive unicystic ameloblastoma in a 7-year-old child: the best approach?. Br J Oral Maxillofac Surg.

[14] Tanaka N, Murata A, Yamaguchi A, Kohama G (1999). Clinical features and management of oral and maxillofacial tumors in children. Oral Surg Oral Med Oral Pathol Oral Radiol Endod.

[15] Fregnani ER, da Cruz Perez DE, De Almeida OP, Kowalski LP, Soares FA, de Abreu Alves F (2010). Clinicopathological study and treatment outcomes of 121 cases of ameloblastomas. Int J Oral Maxillofac Surg.

[16] Nakamura N, Higuchi Y, Tashiro H, Ohishi M (1995). Marsupialization of cystic ameloblastoma: a clinical and histopathologic study of the growth characteristics before and after marsupialization. J Oral Maxillofac Surg.

[17] Scariot R, da Silva RV, da Silva Felix W, da Costa DJ, Rebellato NLB (2012). Conservative treatment of ameloblastoma in child: a case report. Stomatologija.

[18] Tomita Y, Kuroda S, Takahashi T, Ohura R, Tanaka E (2013). Orthodontic occlusal reconstruction after conservative treatment of unicystic ameloblastoma in an adolescent patient: 10-year follow-up. Am J Orthod Dentofac Orthop.

[19] Zhang J, Gu Z, Jiang L, Zhao J, Tian M, Zhou J, Duan Y (2010). Ameloblastoma in children and adolescents. Br J Oral Maxillofac Surg.

[20] Nakamura N, Higuchi Y, Mitsuyasu T, Sandra F, Ohishi M (2002). Comparison of long-term results between different approaches to ameloblastoma. Oral Surg Oral Med Oral Pathol Oral Radiol Endod.

[21] Antonoglou GN, Sándor GK (2015). Recurrence rates of intraosseous ameloblastomas of the jaws: a systematic review of conservative versus aggressive treatment approaches and meta-analysis of non-randomized studies. J Craniomaxillofac Surg.

[22] Furuki Y, Fujita M, Mitsugi M, Tanimoto K, Yoshiga K, Wada T (1997). A radiographic study of recurrent unicystic ameloblastoma following marsupialization. Report of three cases. Dentomaxillofac Radiol.

[23] Dolanmaz D, Etoz OA, Pampu A, Kalayci A, Gunhan O (2011). Marsupialization of unicystic ameloblastoma: a conservative approach for aggressive odontogenic tumors. Indian J Dent Res.

[24] Soliman MM, Abd El Dayem Hassan H, Elgazaerly H, Sweedan TO (2013). Marsupialization as a treatment modality of large jaw cysts. WASJ.

[25] Castro-Núñez J (2016). An innovative decompression device to treat odontogenic cysts. J Craniofac Surg.

[26] Hyomoto M, Kawakami M, Inoue M, Kirita T (2003). Clinical conditions for eruption of maxillary canines and mandibular premolars associated with dentigerous cysts. Am J Orthod Dentofac Orthop.

[27] Ertas Ü, Yavuz MS (2003). Interesting eruption of 4 teeth associated with a large dentigerous cyst in mandible by only marsupialization. J Oral Maxillofac Surg.

